# Comparative Evaluation of Shear Bond Strength: 3D-Printed Composite Versus Zirconia and E-max on Feldspathic Ceramic Rods Using Two Resin Cements

**DOI:** 10.1055/s-0045-1811589

**Published:** 2025-09-23

**Authors:** Mohammed K. Fahmi

**Affiliations:** 1 Department of Restorative Dental Science, Faculty of Dentistry, Taif University, Taif, Saudi Arabia

**Keywords:** zirconia, E-max, 3D-printed composites, shear bond strength, Panavia V5, ResiCem EX, dental restorations

## Abstract

**Objective:**

This article aims to evaluate the shear bond strength of a 3D-printed composite resin compared with well-established materials (zirconia and E-max), bonded with two different resin cements: Panavia V5 and ResiCem EX.

**Materials and Methods:**

Shear bond strength was tested across six material–cement combinations: zirconia, E-max, and 3D-printed composite resin, each bonded with either Panavia V5 or ResiCem EX. A total of 24 discs were prepared from each material, with 12 specimens allocated to each group (10 tested bond strength and 2 microscopy). The bonding interfaces were examined using a digital optical microscope. Shear bond strength was measured using an Instron universal testing machine, and statistical analysis was performed using one-way and two-way ANOVA.

**Results:**

The highest shear bond strength was observed in 3D-printed composite resin bonded with Panavia V5 (20.74 MPa), which was significantly higher than zirconia bonded with ResiCem EX (13.9 MPa, *p* = 0.010). No significant differences were noted between the remaining material–cement combinations.

**Conclusion:**

3D-printed composite resin demonstrated superior bond strength compared with zirconia and E-max; Panavia V5 showed potential as a reliable cement for clinical applications. These findings support the growing role of 3D-printed composites in restorative dentistry.

## Introduction

Restorative dentistry aims to restore both the aesthetics and function of damaged or missing teeth, significantly enhancing oral health and quality of life. Successful restorations depend on durable materials that bond effectively to tooth structures. Unlike provisional restorations, permanent restorations must withstand continuous mechanical forces such as mastication and occlusal loading, necessitating the use of resin cement for secure, long-term adhesion. 1 2 Traditional materials such as IPS Empress ceramics and zirconia offer strong mechanical properties but present limitations, including cracking and inconsistent bonding. 3 According to ISO standards, dental restorations must achieve a minimum bond strength of 5 MPa. IPS e.max typically exhibits bond strengths ranging from 20 to 40 MPa, while zirconia ranges from 10 to 25 MPa. These limitations have spurred interest in alternative materials such as 3D-printed composites, which offer cost-effective solutions and enhanced performance characteristics. 4 5 6

The microstructure of 3D-printed composites—characterized by uniformly distributed ceramic fillers and layered architecture—is essential to mechanical performance, including bond strength, wear resistance, and durability. Interactions between the fillers, matrix–filler interface, and interlayer adhesion directly influence bonding with resin cements, which is essential for successful restorations. 7 8 Properties like filler content, interlayer bonding, and surface texture significantly enhance wear resistance, fracture toughness, and esthetics. 9 Reducing porosity and optimizing cross-link density improve the material's long-term stability. Well-distributed fillers and strong interlayer adhesion also increase surface roughness, promoting mechanical interlocking and chemical bonding with adhesives, thereby enhancing restoration reliability and longevity. 8 9

3D-printed composite resins offer notable advantages for both temporary and permanent dental restorations. In provisional applications, they enable rapid fabrication and customization, allowing clinicians to provide immediate solutions while definitive prosthetics are being prepared. Their adaptability also allows for easy adjustments and replacements. 10 For permanent restorations, including fixed dentures, crowns, inlays, onlays, and veneers, these composites provide precise fit, customization, and improved esthetics. However, their success depends on factors such as material strength, bonding capability, biocompatibility, and long-term durability. Assessing the composite's properties, manufacturing methods, and supporting clinical evidence is essential before adoption in permanent treatments. 11

A key challenge in the clinical application of 3D-printed composite resins is achieving reliable adhesion to tooth structures. Effective bonding depends on proper surface treatment, suitable resin cement selection, and precise light-curing protocols. Addressing these factors requires the use of advanced adhesive systems and standardized clinical procedures. However, there remains a lack of comparative data on the bonding performance of 3D-printed composites relative to conventional ceramic materials, highlighting the need for further research. 11 12

In this study, Vita Mark II feldspathic ceramic rods were chosen due to their uniform mechanical and surface properties, providing a consistent platform for evaluating adhesive interactions in a controlled in vitro setting. This decision was made to prioritize standardization over biological similarity, as our aim was to compare material performance under controlled conditions. While we acknowledge that these substrates do not fully replicate the natural diversity of tooth structure, they minimize variability and allow for standardized comparisons among materials. Future studies will incorporate natural tooth substrates to enhance clinical relevance.

The study aimed to comparatively evaluate the shear bond strength of a 3D-printed composite resin versus zirconia and IPS e.max, using feldspathic ceramic rods as a standardized bonding substrate. Two resin cements—Panavia V5 and ResiCem EX—were employed to assess differences in adhesive performance across material–cement combinations. The findings are intended to advance the understanding of bonding interactions and guide the selection of materials and protocols for durable restorative applications.

The null hypothesis of this study was that there would be no statistically significant difference in shear bond strength among the tested restorative materials—3D-printed composite resin (TriniQ BEGO), zirconia (Ceramill Zolid), and IPS e.max CAD—when bonded with either Panavia V5 or ResiCem EX resin cements.

## Materials and Methods

The study evaluated the performance of four dental materials: VITA YZ TWhite translucent zirconia blocks (LOT 95520), IPS e.max CAD blocks (LOT YB9446), Rodin Sculpture 2 composite resin (LOT 312065), and Vita Mark II blocks (LOT 91260). Two resin cements—Panavia V5 (LOT 000149) and ResiCem EX (LOT 052312)—were used for bonding, as summarized in Table 1 . Zirconia and E-max were included as clinically established materials to serve as internal benchmarks or controls for comparing the bonding performance of the 3D-printed composite resin.

**Table 1:** Materials used for shear bond strength evaluation: material type, commercial name, manufacturer, and lot number

Material type	Commercial name	Manufacturer	Lot number
YZ zirconia	VITA YZ T White translucent zirconia shade white blocks	VITA Zahnfabrik, Bad Säckingen, Germany	95520
IPS e.max	IPS e.max CAD CEREC/inLab blocks	Ivoclar Vivadent, Schaan, Liechtenstein	YB9446
3D-printed composite	Rodin Sculpture 2 Shade A3	DENTCA Inc., Los Angeles, CA, USA	312065
Feldspathic ceramic rod	VITA Mark II block	VITA Zahnfabrik, Bad Säckingen, Germany	91260
Resin cement	Panavia V5	Kuraray Noritake Dental Inc., Tokyo, Japan	000149
Resin cement	ResiCem EX	Shofu Inc., Kyoto, Japan	052312

**Fig. 1:**
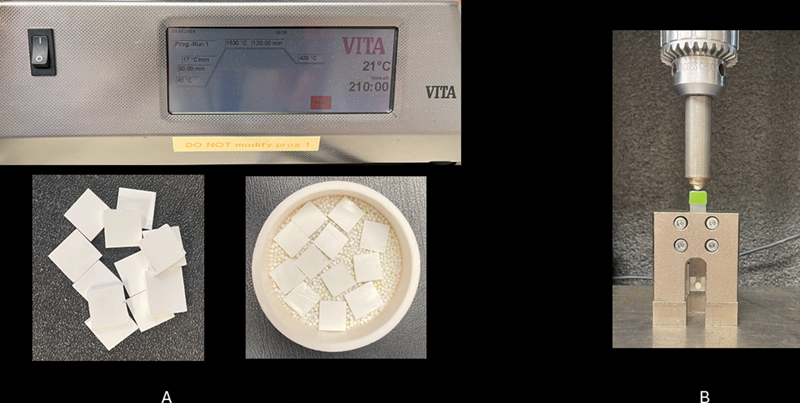
Sample preparation and shear bond strength test of VITA YZ zirconia.

VITA YZ TWhite translucent zirconia shade white blocks (LOT 95520) were sectioned into 24 discs ∼2 mm thick using a Buehler Isomet-2000 precision saw. Sectioning was performed under running water, with the saw operating at 800 rpm and a load of 100 g. A sectional increment of 2.6 mm was used to account for the 0.5 mm blade thickness. After cutting, excess material on the disc edges was removed using 800-grit sandpaper ( Fig. 1A ). The discs were then sintered following the manufacturer's instructions ( Fig. 1A ). Prior to sintering, the specimens measured ∼14 mm × 12 mm × 2 mm. Post-sintering, their dimensions reduced to ∼10.5 mm × 9.6 mm × 1.6 mm, reflecting a shrinkage of ∼25% due to densification. Following sintering, the zirconia discs were air-abraded with 50-μm aluminum oxide particles at 2.5 bar for 10 seconds, from a distance of 10 mm. This surface treatment aimed to increase surface roughness and enhance mechanical retention for improved bonding. Specimens were thoroughly cleaned to remove any residual particles before further processing.

**Fig. 2:**
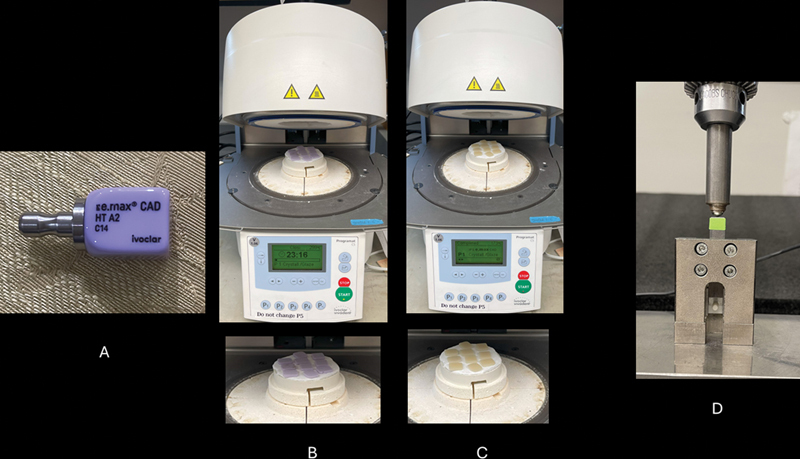
Sample preparation and shear bond strength test of E-max.

IPS E-max CAD blocks (LOT YB9446; Fig. 2A ) were sectioned into 24 discs, each measuring ∼14.5 mm × 12.4 mm × 2.5 mm, using a Buehler Isomet-2000 precision saw. The sectioning was performed under running water, with the saw operating at a blade speed of 800 rpm and a load of 100 g. A sectional increment of 2.6 mm was used to achieve a final disc thickness of 2 mm, accounting for the 0.5 mm saw blade thickness. After sectioning, excess material on the disc edges was removed using 800-grit sandpaper. The discs were then sintered according to the manufacturer's instructions ( Fig. 2B, C ). Following crystallized, the IPS E-max discs were etched with 9.6% hydrofluoric acid Etch Gel (Pulpdent, Lot 161115) for 20 seconds. This etching process selectively dissolved the glassy phase of the ceramic, creating a roughened surface to enhance micromechanical interlocking and chemical bonding with the resin cement. After etching, the discs were thoroughly rinsed with water and air-dried. A silane coupling agent was then applied to further enhance the chemical adhesion between the etched surface and the resin cement.

**Fig. 3:**
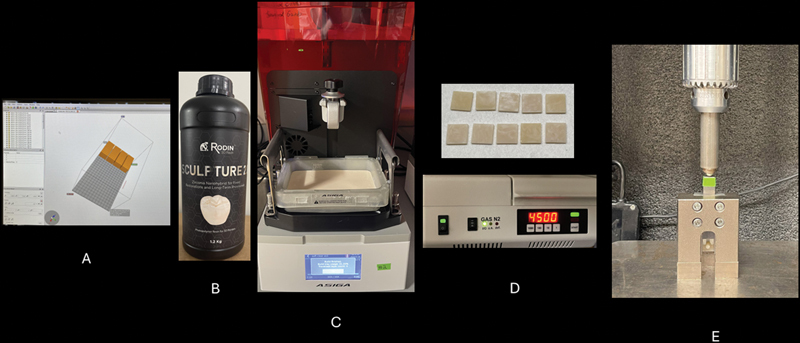
Sample preparation and shear bond strength test of 3D-printed composite resin.

Twenty-four composite resin discs were designed using ASIGA Composer software with dimensions of 14 mm in length, 14 mm in width, and 2 mm in thickness ( Fig. 3A ). The specimens were fabricated using RODIN SCULPTURE 2 Shade A3 (Lot #312065) on an ASIGA 3D printer ( Fig. 3B, C ). After printing, the specimens were cleaned in isopropyl alcohol to remove any uncured resin residues. Final polymerization was performed in a nitrogen gas environment using a dedicated post-curing unit set to 4,500 mW/cm ^2^ , ensuring complete curing and minimizing oxygen inhibition ( Fig. 3D ). This process enhanced the mechanical integrity and surface quality of the printed composite plates

**Fig. 4:**
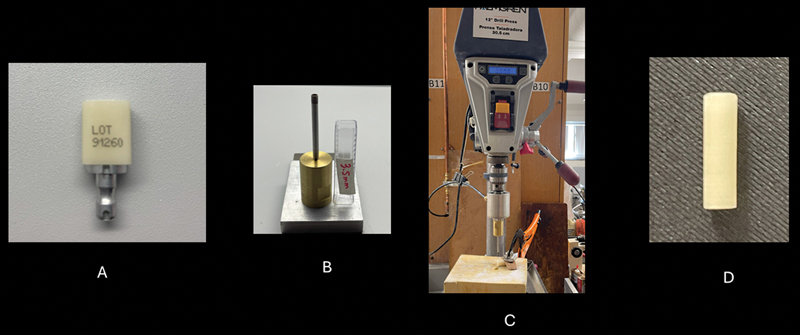
Mark II Rods preparation.

Preparation of Mark II rods: The Vita Mark II block (Lot #91260) ( Fig. 4A ) was securely mounted onto the drill press bed to ensure precise and stable drilling. A 3.5-mm diamond-coated drill bit ( Fig. 4B ), selected for its ability to cut ceramic materials without inducing micro-fractures, was employed. Drilling was conducted at a controlled low speed to prevent overheating and minimize thermal stress, which could otherwise lead to structural damage. Continuous water flow was used as a coolant to maintain optimal temperature for both the drill bit and ceramic block. Steady, consistent pressure was applied throughout the process to avoid chipping or cracking, and the rods were carefully extracted upon completion ( Fig. 4C ). Each ceramic rod was fabricated with an approximate diameter of 3.5 mm and polished to a standardized height of 8 mm ( Fig. 4D ).

After drilling, the 72 ceramic rods were smoothed using fine-grit sandpaper to eliminate any surface irregularities or sharp edges. This was followed by a careful polishing process to achieve a consistent, glossy finish. These steps were essential to standardize the surface characteristics of the rods, ensuring uniformity for accurate bonding and mechanical testing in the subsequent phases of the study ( Fig. 4D ).

After completing the fabrication and surface treatment of all test specimens—including zirconia, IPS E-max, and 3D-printed composite resin discs, as well as the Mark II ceramic rods—each specimen was inspected according to standardized inclusion and exclusion criteria. Specimens were included if they showed uniform dimensions, intact structure, smooth surfaces, and successful surface treatment (sintering, etching, or air abrasion) without visible defects. Specimens were excluded if they displayed any cracks, chips, surface contamination, dimensional inaccuracies, incomplete polymerization (in the case of 3D-printed samples), or improper surface treatment. Only specimens meeting these criteria were selected for sample preparation and included in the bonding and testing procedures.

### Sample Preparation

Discs were fabricated from three restorative materials: IPS e.max CAD, zirconia, and 3D-printed composite resin. A total of 72 disc specimens were prepared and divided into six experimental groups ( *n* = 12 per group) based on the combination of restorative material and resin cement (Panavia V5 [Lot #000149] or ResiCem EX [Lot #052312]). Within each group, 10 specimens were designated for shear bond strength testing, while 2 additional specimens were allocated for scanning electron microscopic (SEM) evaluation of the bonded interface ( Tables 1 and 2 ). Each disc specimen was bonded to a standardized Vita Mark II feldspathic ceramic rod using the assigned resin cement. A vertical load of 500 g (∼5 N) was applied to each specimen for 5 minutes using a custom-made weight apparatus to ensure uniform pressure during cementation. This standardized protocol was adopted to ensure consistent cement thickness and minimize operator-induced variability across all groups.

**Table 2:** Specimen allocation and grouping details

Group	Subgroup	Description	No. of specimens	Note on examination
Group A	Group A1	YZ Zirconia + Panavia V5 cements	12	10 for bond strength, 2 for microscopy
	Group A2	YZ Zirconia + ResiCem EX resin cements	12	10 for bond strength, 2 for microscopy
Group B	Group B1	E-max + Panavia V5 cements	12	10 for bond strength, 2 for microscopy
	Group B2	E-max + ResiCem EX resin cements	12	10 for bond strength, 2 for microscopy
Group C	Group C1	3D-printed composite + Panavia V5 cements	12	10 for bond strength, 2 for microscopy
	Group C2	3D-printed composite + ResiCem EX resin cements	12	10 for bond strength, 2 for microscopy

To enhance adhesion between the ceramic rods and the discs, a ceramic primer was applied to the surface of the Vita Mark II rods prior to the application of resin cement. The primer was used according to the manufacturer's instructions to promote optimal chemical bonding. After primer application, the resin cement was applied, and the bonded assemblies were cured under standardized laboratory conditions, including ambient room temperature (∼23 ± 1°C).

Light-curing was performed using an LED curing unit (Bluephase G2, Ivoclar Vivadent) with an intensity of 1,200 mW/cm ^2^ for 20 seconds from two opposite sides of the specimen surface to ensure complete polymerization.

All bonded specimens were stored in distilled water at 37°C for 24 hours prior to shear bond strength testing.

Shear bond strength was evaluated for all groups using a half-round blade. Ten specimens from each group were tested. The blade, featuring a 4-mm diameter and a 2-mm edge thickness, was centrally aligned to ensure precise contact with the bonded interface ( Fig. 1B , Fig. 2D , Fig. 3E ). Testing was conducted on an Instron universal testing machine (Model 5566A), operated at a crosshead speed of 0.5 mm/min and equipped with a 1-kN load cell. Each specimen was securely mounted in a shear bond testing fixture, with the blade positioned perpendicular to the interface between the Vita Mark II rods and their respective substructures (zirconia, E-max, and 3D-printed composite resin). During testing, the load was applied parallel to the adhesive interface. Shear bond strength was calculated in megapascals (MPa) by dividing the load at failure by the cross-sectional area of the bonded interface, using the formula: shear bond strength (MPa) = F/ *πr* 2, where *F* is the load at failure (in newtons), *r* is the radius of the bonded area (in millimeters), and *π* is ∼3.14. 13

Fractured specimens were examined under a stereomicroscope (25– × 40 magnification), and failure modes were classified as adhesive, cohesive, or mixed. Residual resin cement on the fractured surfaces was used to aid in determining the failure type after bonding to feldspathic ceramic rods.

This protocol was developed based on standard laboratory procedures commonly used in previous studies involving shear bond strength testing of ceramic and resin materials. Where applicable, procedural details were adapted and optimized for the current study context using feldspathic ceramic rods.

Two specimens from each group were selected for interface evaluation between the resin cements (Panavia V5 and ResiCem EX) and the tested restorative materials (zirconia, IPS e.max CAD, 3D-printed composite resin, and Mark II rods). Digital optical microscopy was used to examine the bonded interfaces, with particular focus on interfacial adaptation and bonding consistency across material combinations. Although not a primary study variable, cement layer thickness was qualitatively assessed to identify intra- and intergroup variability observed during microscopic examination.

A two-way ANOVA was conducted to analyze differences in shear bond strength across three materials (zirconia, E-max, and 3D-printed composite resin) and two types of resin cement (Panavia V5 and ResiCem EX). This statistical method was used to assess the main effects of material type and cement type, as well as any interaction effects between these factors on shear bond strength. Additionally, a one-way ANOVA was performed to compare shear bond strength among the six material–cement combinations. When significant differences were detected in the one-way ANOVA, post-hoc Tukey HSD tests were applied to identify specific group differences. The significance level for all analyses was set at *p* < 0.05, and all tests were conducted using SPSS software.

## Results

The effects of material type (zirconia, E-max, and 3D-printed composite resin) and resin cement (Panavia V5 and ResiCem EX) on shear bond strength were evaluated using one-way and two-way ANOVA.

A one-way ANOVA was performed to compare shear bond strength among six material–cement groups: zirconia with Panavia V5, zirconia with ResiCem EX, E-max with Panavia V5, E-max with ResiCem EX, 3D-printed composite resin with Panavia V5, and 3D-printed composite resin with ResiCem EX. The analysis revealed a statistically significant effect of the material–cement combination on bond strength ( *F* (5, 54) = 3.132, *p* = 0.015), indicating that both material and cement types influence bond strength ( Table 3 ). Tukey's post hoc test showed that 3D-printed composite resin bonded with Panavia V5 had significantly higher bond strength than zirconia bonded with ResiCem EX (mean difference = 10.45, *p* = 0.010) ( Table 4 ). Although 3D-printed composite resin with Panavia V5 also demonstrated greater bond strength compared with zirconia with Panavia V5, E-max with Panavia V5, and E-max with ResiCem EX, these differences were not statistically significant ( *p* > 0.05). No significant difference was found between 3D-printed composite resin bonded with Panavia V5 and with ResiCem EX ( Table 4 ). These findings suggest that the superior performance of 3D-printed composite resin bonded with Panavia V5—especially when compared with zirconia with ResiCem EX—was the primary contributor to the observed differences in bond strength. However, since other material–cement combinations did not exhibit significant differences, it suggests that bond strength is more strongly influenced by the material type than by the resin cement. A two-way ANOVA was then conducted to examine the individual effects of material type (zirconia, E-max, and 3D-printed composite resin) and resin cement type (Panavia V5 and ResiCem EX), as well as their interaction, on shear bond strength ( Table 5 ). The analysis demonstrated a statistically significant main effect of material type ( *F* (2, 54) = 5.572, *p* = 0.006), with the 3D-printed composite resin exhibiting significantly higher bond strength compared with both zirconia and E-max. However, there was no significant difference between zirconia and E-max. The type of resin cement did not have a statistically significant effect on bond strength across all groups (F(1, 54) = 3.468, *p* = 0.068). Furthermore, no significant interaction was found between material type and resin cement type (F(2, 54) = 0.523, *p* = 0.596), indicating that the influence of material type on bond strength was consistent regardless of the cement used.

**Table 3:** ANOVA summary for shear bond strength

Source	Sum of squares	df	Mean square	*F* -value	*p* -Value
Between groups	675.963	5	135.193	3.132	0.015
Within groups	2,331.243	54	43.171		
Total	3,007.206	59			

**Table 4:** Tukey HSD post hoc comparisons for shear bond strength

Comparison groups	*p* -Value
Zirconia Panavia V5 vs. zirconia ResiCem EX	0.706
Zirconia Panavia V5 vs. E-max Panavia V5	0.994
Zirconia Panavia V5 vs. E-max ResiCem EX	0.967
Zirconia Panavia V5 vs. 3D-printed composite resin Panavia V5	0.291
Zirconia Panavia V5 vs. 3D-printed composite resin ResiCem EX	0.992
Zirconia ResiCem EX vs. E-max Panavia V5	0.949
Zirconia ResiCem EX vs. E-max ResiCem EX	0.988
Zirconia ResiCem EX vs. 3D-printed composite resin Panavia V5	0.010 a
Zirconia ResiCem EX vs. 3D-printed composite resin ResiCem EX	0.351
E-max Panavia V5 vs. E-max ResiCem EX	1.000
E-max Panavia V5 vs. 3D-printed composite resin Panavia V5	0.097
E-max Panavia V5 vs. 3D-printed composite resin ResiCem EX	0.868
E-max ResiCem EX vs. 3D-printed composite resin Panavia V5	0.055
E-max ResiCem EX vs. 3D-printed composite resin ResiCem EX	0.745
3D-printed composite resin Panavia V5 vs. 3D-printed composite resin ResiCem EX	0.635

a Statistically significant at *p* < 0.05.

**Table 5:** Two-way ANOVA summary for shear bond strength (tests of between-subjects effects, dependent variable: shear bond strength

Source	Type III sum of squares	df	Mean square	F-value	*p* -Value
Corrected model	675.963 a	5	135.193	3.132	0.015
Intercept	19,589.177	1	19,589.177	453.756	<0.001
Material	481.096	2	240.548	5.572	0.006
Resin cement	149.705	1	149.705	3.468	0.068
Material * resin cement	45.163	2	22.581	0.523	0.596
Error	2,331.243	54	43.171		
Total	22,596.383	60			
Corrected total	3,007.206	59			

a R-squared = 0.225 (adjusted R-squared = 0.153).

Descriptive statistics offered further insight into the bond strength performance of each material–cement combination. The 3D-printed composite resin paired with Panavia V5 exhibited the highest mean shear bond strength (mean = 20.74 MPa, SD = 6.94), whereas zirconia combined with ResiCem EX showed the lowest value (mean = 13.9 MPa, SD = 4.99). These findings highlight the superior performance of the 3D-printed composite resin, especially when used with Panavia V5. Microscopic evaluation of the bonding interfaces revealed no major visual differences in bonding characteristics among the materials (zirconia, E-max, 3D-printed composite resin, and Mark II rods) or between the resin cements (Panavia V5 and ResiCem EX).

Fracture analysis revealed that the majority of specimens exhibited adhesive failure, with separation consistently occurring at the interface between the restorative materials—zirconia, IPS e.max CAD, and 3D-printed composite resin—and the VITA Mark II feldspathic ceramic rods. In all cases, resin cement remnants remained predominantly on the ceramic rod surface, indicating that failure occurred at the restorative material–cement interface. Minor cement residues were occasionally observed on the restorative surfaces, suggesting a limited cohesive component within the resin cement. No cohesive failure was observed within the ceramic rods or the restorative materials.

**Fig. 5:**
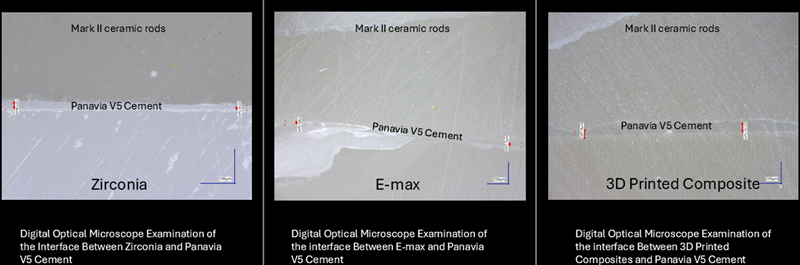
Digital optical microscope images showing the interface between Mark II ceramic rods and three restorative materials (zirconia, E-max, and 3D-printed composite resin) bonded with Panavia V5 cement. The cement layer is clearly visible between the ceramic rods and each material. Variations in cement thickness are evident across and within groups. Scale bars: zirconia (100 µm), E-max (250 µm), 3D-printed composite (150 µm).

As shown in Fig. 5 , variability in cement layer thickness was noted both within individual specimens and among the experimental groups. Although cement thickness was not a study variable and was not deliberately manipulated, such inconsistencies may have contributed to the broader range of standard deviations observed in the shear bond strength values. In certain cases, thickness variation was evident even within a single specimen, potentially contributing to the dispersion of bond strength outcomes without significantly affecting the mean values.

In summary, the type of restorative material had a significant impact on shear bond strength, with 3D-printed composite resin demonstrating superior performance compared with both zirconia and E-max. Although the choice of resin cement—Panavia V5 or ResiCem EX—did not significantly affect bond strength across all materials, the combination of 3D-printed composite resin and Panavia V5 produced the highest mean bond strength among all tested groups.

## Discussion

3D-printed composite resins are gaining increasing attention as promising materials for dental restorations, primarily due to their customizable properties and the precision offered by additive manufacturing techniques. 14 15 16 In this study, Mark II ceramic rods were used to simulate tooth structure during shear bond strength testing. This provided a consistent and standardized substrate, enhancing the reliability of comparisons among the different restorative materials. The use of Mark II rods eliminated variability commonly associated with natural teeth, such as differences in dentin composition, microstructure, and morphology, which can significantly affect bonding performance. 17 By employing a uniform substrate, we ensured that the observed differences in bond strength were attributable to the restorative materials and resin cements rather than inconsistencies in the tooth analog. 17 The use of zirconia and E-max as reference materials allowed for a meaningful evaluation of the 3D-printed composite resin's potential for future applications beyond temporary restorations.

While this design prioritizes standardization over clinical mimicry, we acknowledge that natural tooth substrates may offer greater clinical relevance. Therefore, future studies are recommended to validate these findings under more clinically representative conditions.

The results demonstrated that the type of restorative material had a significant effect on shear bond strength to the Mark II ceramic substrate, with 3D-printed composite resin exhibiting superior performance compared with both zirconia and IPS E-max. This enhancement is likely due to the unique microstructure of the 3D-printed composite, which facilitates improved mechanical interlocking and chemical adhesion with resin cement. 16 18 The additive manufacturing process, characterized by its layer-by-layer fabrication, generates a microtextured surface that increases the available bonding area, potentially enhancing adhesion to the Mark II rods. 19

The polymer matrix of the 3D-printed composite resin may exhibit a higher chemical affinity with resin cements, promoting interdiffusion and chemical bonding at the material–cement interface. 2 17 This chemical compatibility promotes more effective coupling between the restorative material and the adhesive system, ultimately improving bond strength. Additionally, the inclusion of nano-sized ceramic fillers enhances the composite's mechanical properties and wear resistance, further supporting its suitability for definitive dental restorations. 20

In contrast, bonding to traditional ceramics such as zirconia and IPS E-max remains more complex due to their chemically inert surfaces and low surface energy, even when bonded to standardized ceramic substrates like Mark II rods. Achieving reliable adhesion with these materials typically requires specific surface treatments to enhance mechanical and chemical retention. Zirconia is commonly subjected to sandblasting to generate surface roughness and improve micromechanical retention, whereas IPS E-max is etched with hydrofluoric acid (HF) to increase surface irregularities and facilitate chemical bonding with resin cements. 21 Nevertheless, despite these treatments, zirconia and IPS E-max generally exhibit lower bond strength values compared with resin-based materials such as 3D-printed composite resins, which benefit from both enhanced mechanical interlocking and chemical bonding due to their distinctive microstructure.

The absence of a statistically significant difference between Panavia V5 and ResiCem EX in terms of bond strength suggests that, when bonding to standardized ceramic substrates like Mark II rods, the selection of resin cement may be secondary to the choice of restorative material. From a clinical standpoint, this finding provides flexibility, allowing clinicians to select resin cements based on preferences such as handling characteristics, esthetics, or cost—without compromising bonding performance. However, it is important to recognize that these results may not fully extend to natural tooth structures. The complex structure of dentin and its variable interaction with different resin cements may significantly influence bond strength outcomes. Therefore, further studies are necessary to validate these findings across a broader range of clinical substrates and conditions. 17

The shear bond strength values observed for zirconia and IPS E-max in this study were generally consistent with previously reported ranges, with zirconia exhibiting bond strengths between 13.9 and 18.11 MPa, and IPS E-max ranging from 15.77 to 16.49 MPa. Discrepancies in the literature can be attributed to several factors, including variations in the composition of the core materials, differences in surface treatment techniques—such as sandblasting for zirconia and hydrofluoric acid etching for IPS E-max—and the specific type of resin cement utilized. Furthermore, inconsistencies in specimen preparation methods, curing protocols, and testing procedures across studies can influence the reported outcomes. The intrinsic microstructure of each material also plays a pivotal role: the crystalline nature of zirconia typically necessitates mechanical retention, whereas the glass-ceramic matrix of IPS E-max is more amenable to chemical bonding following etching, both of which critically affect bond strength performance. 21 22 23 24 25 26 27

In this study, 3D-printed composite resins exhibited shear bond strengths ranging from 16.89 to 20.74 MPa, aligning with the broad spectrum of previously reported values, which range from 8 to 38 MPa. 11 28 While our results surpassed some prior findings and fell below others, this variability is likely attributable to differences in composite formulations, surface treatment protocols, and experimental methodologies. Notably, 3D-printed composites exhibited bond strengths comparable to or even exceeding those of conventional restorative materials such as zirconia and IPS e.max. 21 23 24 26 27 These findings underscore the potential of 3D-printed materials for durable clinical applications. However, continued research is necessary to refine surface treatment techniques to further enhance adhesion performance. In conclusion, the evolving capabilities of 3D printing technology offer promising opportunities in restorative dentistry, but surface optimization remains essential for maximizing bond strength.

Variation in cement thickness, as revealed by interface analysis, may have contributed to the wide range and high standard deviations observed in shear bond strength values. While cement thickness was not a primary variable in this study, inconsistencies—both within and between specimen groups—were noted. Thicker cement layers are known to increase the risk of voids or uneven stress distribution, while thinner layers (typically 50–150 µm) are generally associated with more favorable stress transfer and improved adhesion in the literature. Although these differences did not appear to significantly influence the mean bond strength in our study, they may explain the broader distribution of values and the relatively high standard deviations observed. These findings suggest that improved control of cement thickness may help reduce variability in adhesive performance assessments. 29 30

In summary, this study evaluated the shear bond strength of 3D-printed composite resin, zirconia, and IPS e.max using Panavia V5 and ResiCem EX resin cements. The 3D-printed composite resin demonstrated significantly higher bond strength compared with zirconia and IPS e.max, while the type of resin cement had no significant effect—indicating clinical flexibility in cement selection. Microscopic analysis showed that variations in cement layer thickness contributed to inconsistencies in bond strength measurements.

As this was an in vitro investigation, the findings should be interpreted with caution regarding their direct clinical applicability. Further in vivo studies are necessary to evaluate performance under functional and biological conditions. A key limitation of this study was the variability in cement thickness, which—despite efforts to standardize the procedure—may have influenced the bond strength outcomes. Although the sample size of 10 specimens per group is commonly used in similar in vitro studies, the lack of a priori power analysis is acknowledged as a limitation that may affect the generalizability of the findings.

While the superior bonding performance of 3D-printed composite resin is encouraging, these findings should be interpreted cautiously within the limitations of this in vitro investigation. The results indicate that additive manufacturing holds promise as a viable alternative to conventional CAD/CAM ceramics, especially in digitally driven restorative workflows. However, to ensure clinical reliability and long-term performance, further in vivo studies are essential. Future research should prioritize the optimization of surface treatment protocols, evaluation of bonding behavior on natural tooth substrates, and improvement of mechanical properties for use in high-stress intraoral environments.

## Conclusion

Within the limitations of this in vitro study, 3D-printed composite resin demonstrated significantly higher shear bond strength compared with zirconia and IPS e.max when bonded to a standardized ceramic substrate. These results indicate that the type of restorative material may play a more significant role in bond strength outcomes than the choice of resin cement, as no statistically significant differences were found between Panavia V5 and ResiCem EX. While the findings highlight the potential of additive manufacturing in restorative dentistry, further investigations—including both extended in vitro experiments and well-designed in vivo studies—are needed to evaluate long-term performance, optimize surface treatment protocols, and assess bonding behavior on natural tooth structures under clinical conditions.
